# Secondhand smoke exposure and mental health problems in Korean adults

**DOI:** 10.4178/epih.e2016009

**Published:** 2016-03-14

**Authors:** Na Hyun Kim, Hansol Choi, Na Rae Kim, Jee-Seon Shim, Hyeon Chang Kim

**Affiliations:** 1 Department of Public Health, Yonsei University Graduate School, Seoul, Korea; 2 Cardiovascular and Metabolic Diseases Etiology Research Center, Yonsei University College of Medicine, Seoul, Korea; 3 Department of Preventive Medicine, Yonsei University College of Medicine, Seoul, Korea

**Keywords:** Secondhand smoke, Mental health, Depression, Stress

## Abstract

**OBJECTIVES:**

To evaluate the association between secondhand smoke exposure (SHSE) and mental health problems among Korean adults.

**METHODS:**

We analyzed data from the 2011 Korean Community Health Survey. From the total of 229,226 participants aged 19 years or above, we excluded 48,679 current smokers, 36,612 former smokers, 3,036 participants with a history of stroke, 2,264 participants with a history of myocardial infarction, 14,115 participants who experienced at least one day in bed per month due to disability, and 855 participants for whom information regarding SHSE or mental health problems was not available. The final analysis was performed with 22,818 men and 100,847 women. Participants were classified into four groups according to the duration of SHSE: none, <1 hr/d, 1-<3 hr/d, and ≥3 hr/d. The presence of depressive symptoms, diagnosed depression, and high stress were measured by questionnaire.

**RESULTS:**

After adjusting for demographic factors, lifestyle, and chronic disease, the odds ratio (OR) and 95% confidence interval (CI) of depressive symptoms with 1-<3 hr/d and ≥3 hr/d SHSE were 1.44 (95% CI, 1.14 to 1.82) and 1.59 (95% CI, 1.46 to 1.74), respectively. However, SHSE ≥3 hr/d had a higher OR of 1.37 (95% CI, 1.20 to 1.58) for diagnosed depression. SHSE was also associated with high stress (1-<3 hr/d: OR, 1.56; 95% CI, 1.38 to 1.76; ≥3 hr/d: OR, 1.33 95% CI, 1.28 to 1.40). However, the association between SHSE and symptoms of depression and stress did not differ significantly by region.

**CONCLUSIONS:**

SHSE may be associated with mental health problems such as depression and stress in Korean adults.

## INTRODUCTION

Around 58 million non-smokers are exposed to secondhand smoke, according to the Centers for Disease Control and Prevention (CDC) [1], and even a low level of secondhand smoke exposure (SHSE) causes significant health problems [2]. SHSE is associated with inflammation and cardiovascular disease [3,4]. There is also evidence for a detrimental effect of SHSE on mental health in adults [5-9], although some studies do not support this [10,11]. Mental illness of extended duration or moderate or severe intensity leads to significant morbidity and reduced daily function, and may be associated with suicide, the incidence of which is increasing in Korea.

The purpose of the present study was to explore the association between SHSE and symptoms of depression and stress in Korean adults who had never been cigarette smokers themselves, using data from the Korean Community Health Survey (KCHS). Our previous studies have reported a positive association between SHSE and mental health problems; however, these studies focused only on depression [12,13]. In the present study, we hypothesized that SHSE was associated with mental health problems including depressive symptoms, diagnosed depression, and high stress.

## MATERIALS AND METHODS

### Study population

This study is a cross-sectional analysis from the 2011 KCHS organized by the Korea Centers for Disease Control and Prevention (KCDC). The survey enrolled 229,226 adults aged 19 or older from August to October 2011. From the total 229,226 participants aged 19 years or above, we excluded 48,679 current smokers, 36,612 former smokers, 3,036 participants with a history of stroke, 2,264 participants with a history of myocardial infarction, 14,115 participants who experienced at least one day in bed per month due to disability [14], and 855 participants for whom information regarding SHSE or mental health problems was not available. The final analysis was performed with 22,818 men and 100,847 women. This study was approved by the institutional review board (IRB) of KCDC (IRB no. 2011- 05CON-04-C).

### Measurements

A representative population sample for the KCHS is selected annually based on nationwide address data from the Ministry of Public Administration and Security and on data on housing types and the number of households from the Ministry of Land, Transport and Maritime Affairs. Sample households were extracted from these data with the aim of surveying an average of 900 individuals for each health center [15]. The operating committee, specialized subcommittees, and administration office that conducted the survey were formed through a partnership among the KCDC, 16 cities and provinces, 253 health centers, and 36 universities [15,16].

Participants were individually interviewed in their own household by trained interviewers, who conducted one-on-one surveys using a laptop computer. Information was collected about sociodemographic characteristics, health behaviors, and history of chronic disease. Height and weight were measured, and body mass index (BMI) was calculated as weight in kilograms divided by the square of height in meters. Marital status was categorized into three groups: never married, married, and divorced/separated/widowed. Education level was divided into three groups: middle school or lower, high school, and college or higher. Occupation was categorized into four groups: experts, clerical/services roles, simple skilled roles, and unemployed/homemaker. Monthly income was divided into quartiles: <1.0 million Korean won (KRW), 1.0 -<2.5 million KRW, 2.5 -<4.0 million KRW, and ≥4.0 million KRW. Exercise was described as those who did severe exercise ≥20 minutes for ≥3 times per week, or those who did moderate exercise ≥30 minutes at least once per week. Regular alcohol consumption was defined as more than five glasses of alcohol for two days per week. A history of hypertension, diabetes, or dyslipidemia was classified as the presence of chronic disease. Sleep time was determined in terms of the number of hours slept each day. SHSE was categorized into four groups: none, <1 hr/d, 1-<3 hr/d, and ≥3 hr/d.

### Assessment of symptoms of mental illness

The presence of depressive symptoms were assessed using the question “Have you felt little pleasure or hopeless continuously for over two weeks during the past year, and did this impact on your everyday life?”. The presence of diagnosed depression was assessed using the question “Have you ever been diagnosed with depression by a doctor?”. Perceived stress was measured by asking participants about whether they experienced a level of stress in daily life that was minimal to mild (low stress) or moderate to severe (high stress).

### Statistical analysis

General characteristics were described for a total of 123,665 participants using an independent t-test and a chi-square test. SHSE and other variables were compared between participants with depressive symptoms, diagnosed depression, or high stress and those without these conditions using an ANOVA and a chi-square test. The association between SHSE and mental health problems was assessed using logistic regression models adjusted for age, gender, BMI, marital status, education level, occupation, monthly income, exercise, regular alcohol consumption, and history of chronic disease. We performed a subgroup analysis to assess whether sociodemographic factors modified the association between SHSE and mental health problems.

We estimated the mean, prevalence, and odds ratio (OR) using sampling weights. Statistical analyses were performed using the PROC SURVEY procedure in SAS version 9.4 (SAS Institute Inc., Cary, NC, USA). All analyses were two-sided and p-values less than 0.05 were regarded as statistically significant.

## RESULTS

General characteristics are shown by gender in Table 1. The mean age was significantly higher in women, and the mean BMI was significantly higher in men. Men were more likely to have a higher education level and monthly income, be employed, and to perform regular exercise and be a regular alcohol drinker than women. Women had a higher prevalence of chronic disease than men and a higher prevalence of SHSE (61.2%), depressive symptoms (5.5%), diagnosed depression (2.6%), and high stress (25.7%) than men.

Table 2 presents the prevalence of depressive symptoms, diagnosed depression, and high stress according to participant characteristics. The prevalence of depressive symptoms and diagnosed depression were higher in participants aged 60 to 69 years, divorced/separated/widowed, educated to the level of middle school or lower, and those who were unemployed or homemakers. The prevalence of high stress was higher in participants aged 30 to 39 years, were never married, educated to a college level or higher, and worked as experts or in a clerical/services role. Participants with a low monthly income were more likely to have mental health problems. Participants experiencing SHSE of ≥3 hr/d had a higher prevalence of depressive symptoms (6.2%), while those with SHSE of <1 hr/d were more likely to have diagnosed depression (3.0%). Meanwhile, participants with SHSE of 1-<3 hr/d had a prevalence of high stress (33.9%).

**Figure 1.**
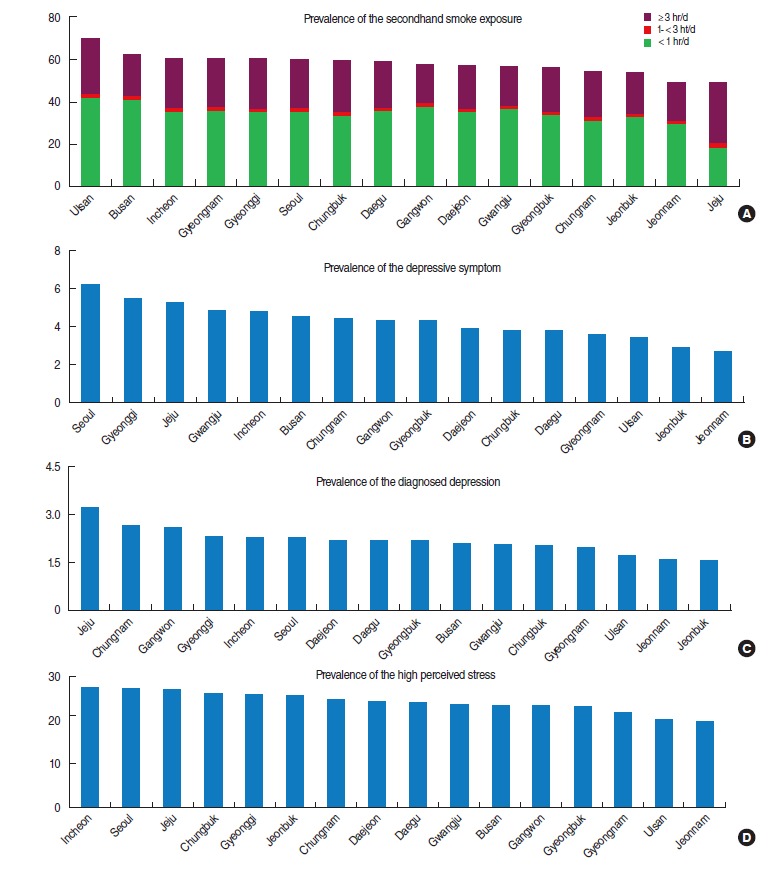
Prevalence of the secondhand smoke exposure (A) and mental health problems (B: depressive symptom, C: diagnosed depression, and D: high perceived stress) by region.

Figure 1 shows that the regional prevalence of SHSE ranged from 49.3% (Jeju) to 70.4% (Ulsan). The prevalence of depressive symptoms ranged from 2.7% (Jeonnam) to 6.2% (Seoul), while the prevalence of diagnosed depression ranged from 1.6% (Jeonbuk) to 3.2% (Jeju). The prevalence of high stress ranged from 19.6% (Jeonnam) to 27.6% (Incheon).

Table 3 describes the association between SHSE and mental health problems in all participants, adjusting for covariates. Compared to participants with no SHSE, those with 1-<3 hr/d SHSE or SHSE of ≥3 hr/d had a higher OR and 95% confidence interval (CI) for depressive symptoms 1.44 (95% CI, 1.14 to 1.82) and 1.59 (95% CI, 1.46 to 1.74), respectively. Participants with SHSE of ≥3 hr/d had an OR of 1.37 (95% CI, 1.20 to 1.58) for diagnosed depression. SHSE was also associated with high stress (1-<3 hr/d: OR, 1.56; 95% CI, 1.38 to 1.76; ≥3 hr/d: OR, 1.33; 95% CI, 1.28 to 1.40). In men, SHSE of 1-<3 hr/d or ≥3 hr/d was significantly associated with depressive symptoms and high stress. In women, SHSE of 1-<3 hr/d or ≥3 hr/d was significantly associated with depressive symptoms and high stress, and women with SHSE ≥3 hr/d had significantly higher odds of diagnosed depression.

**Figure 2.**
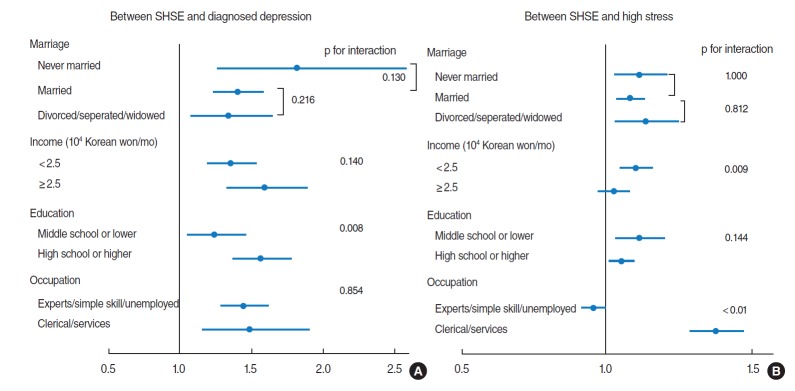
Figure captionAssociation between secondhand smoke exposure (SHSE) and mental health problems (A: diagnosed depression, B: high stress) by sociodemographic factors. Odds ratio and 95% confidence interval adjusted for age, gender, body mass index, marital status, education level, occupation, income, regular exercise, alcohol drinking, hypertension, diabetes, dyslipidemia, sleep time. SHSE was divided into two groups; no SHSE (reference) and SHSE.

We performed a subgroup analysis to assess whether sociodemographic factors modify the association between SHSE and mental health problems. SHSE was associated with marital status, income, education and occupation (Appendix 1). Diagnosed depression was related to income, education, and occupation (Appendix 2), and high stress was associated with marital status, income, education, occupation, and drinking (Appendix 3). Thus, we evaluated the association between SHSE and mental health problems according to these sociodemographic factors (Figure 2).

The association between SHSE and depression was higher in the higher educated group than less educated group (interaction p-value=0.008).

The association between SHSE and high stress was significantly different according to income status and occupation. The association was higher in the two lower income groups (<2.5 million KRW/mo) than the two higher income groups (≥2.5 million KRW/mo).

In the occupational group of clerical/services roles, the OR of high stress with SHSE was higher than in the other occupational groups (data not shown). To facilitate clear interpretation of this interaction, we re-categorized occupation into two groups: clerical/services roles, and other roles (experts, simple skilled roles, and unemployed or homemakers). The relationship between SHSE and high stress was significantly higher in the new clerical/services roles group than the group of other roles.

Regular alcohol consumption was not independently associated with SHSE or diagnosed depression, and did not affect the association between SHSE and mental health problems (data not shown). As performing further analysis, we found that former and current smokers had higher odds of mental health problems compared to non-smokers (Appendix 4).

## DISCUSSION

We observed a positive association between SHSE and depressive symptoms, diagnosed depression, and high stress in a representative sample of Korean adults. Of note, participants with SHSE of more than three hours had higher odds of these mental health problems compared to those with no SHSE. Men with SHSE of less than an hour or more than three hours had higher odds for depressive symptoms and high stress. However, women with more frequent SHSE mostly had higher odds of the mental health problems examined.

To the best of our knowledge, this is the first report on the association between SHSE and mental health problems in Korean adults, although our previous studies reported the association of SHSE with depression [12,13]

Several studies have reported a positive association between SHSE and detrimental effects on mental health. A previous study reported that SHSE, assessed by an objective method, was associated in a dose-dependent manner with psychological distress and risk of future psychiatric illness in a Scottish adult population [5]. Another study based on the US National Health and Nutrition Examination Survey found that SHSE was positively associated with depressive symptoms after adjusting for age, ethnicity, gender, education, and chronic diseases [6]. A recent study also observed that children exposed to secondhand smoke *in utero* and in childhood had an increased risk of depression in midlife, even after adjusting for direct and indirect exposure to tobacco smoke in adulthood [8]. Another US study reported that persistent SHSE was associated with an increased risk of depression and panic attacks [9]. However, some studies have demonstrated a negative association between SHSE and mental health. SHSE was not associated with depression or mental health in the UK Health and Lifestyle Survey [11], and another study also found no association of SHSE with depression and anxiety in data combined from the Netherlands Study of Depression and Anxiety and the Netherlands Twin Register [10].

There are several possible explanations for the association of SHSE with symptoms of depression and stress among adults. First, secondhand smoke itself can be a chronic stressor for non-smokers, leading to worsening mental health [17].

Second, there are several plausible biological mechanisms that may explain the association. The dopaminergic system is involved in the pathogenesis of several mental illnesses, including depression [18], and several animal studies suggest that tobacco smoke has acute and long-term effects on the dopamine system. Exposure to tobacco smoke has been reported to elevate dopamine D1 and D2 receptors [19] and alter the expression of gamma-aminobutyric acid B2 receptors, dopamine transporter messenger RNA, and dopamine receptors in the rat brain [20]. Other animal studies indicate that nicotine and tobacco particulate matter influences long-term imbalances of dopamine transport [21], and most importantly that nicotine exposure induces negative mood and decreased mobility in rats [22].

Another biological mechanism that may link SHSE to mental illness is chronic inflammation [23,24]. Many studies have proposed that the activation of inflammatory cytokines plays a role in mental illnesses [25]. Cytokines induce the enzyme indoleamine 2,3-dioxygenase, which limits tryptophan and serotonin transporters and may thus cause depression [26].

Even though smoke-free legislation has contributed to a successful decline in SHSE, around 10% to 15% of adults continue to be exposed to secondhand smoke at home or workplace [27]. Mental illness imposes an important economic burden, not just on individuals but also on families and communities [28]. Therefore, modifying potential risk factors, for example by monitoring the exposure to secondhand smoke, could not only mitigate the impact of the recession on deaths and injuries arising from suicide, but also reduce economic burden [10].

The present study has several limitations. First, as a cross-sectional study in which all information was gathered at the same point in time, it cannot establish a temporal relationship between SHSE and mental health problems. Second, our use of an interviewer-assisted questionnaire has a limited potential for validation. Therefore, the misclassification bias in measuring SHSE and mental health problems, if any, is likely to be a non-differential reduction of the association. Third, data on other household members who smoked or on sleep quality were not available in KCHS, but may be potential factors that could influence our data. According to previous studies, living with household members who are smokers could be related to SHSE [29], and sleep quality could be closely associated with mental illness [30].

Lastly, our data included only permanent residents of Korea; therefore, our results may not be generalizable to other countries.

In conclusion, our results suggest that SHSE is associated with depressive symptoms, diagnosed depression, and high stress in a dose-dependent manner among Korean adults, regardless of not only the exposure location but also the region. Since we used a large nationwide representative sample, our findings may be generalizable to the general Korean population. We also observed that sociodemographic factors such as household income and occupation might modify the association between SHSE and mental health problems. Further studies are needed to confirm the causal effects of SHSE on the development and aggravation of mental health problems, and to identify the underlying biological mechanisms.

## Figures and Tables

**Table 1.** Basic characteristics by gender

Variables (n = 123,665)	Men (n = 22,818)	Women (n = 100,847)	p-value
Age (yr)	45.7±17.6	49.8±16.3	< 0.001
Body mass index (kg/m2)	23.6±2.9	22.5±3.1	< 0.001
Sleep time (hr)	6.65 ±1.1	6.66±1.2	< 0.001
Age (yr)			< 0.001
19-29	5,460 (36.9)	12,201 (18.3)	
30-39	3,832 (19.1)	17,552 (20.7)	
40-49	4,030 (17.3)	20,681 (22.6)	
50-59	3,778 (13.9)	21,098 (19.2)	
60-69	3,098 (7.4)	15,491 (11.0)	
≥70	2,620 (5.4)	13,824 (8.2)	
Marital status			< 0.001
Never married	6,907 (42.9)	12,504 (18.2)	
Married	14,676 (53.4)	68,609 (66.2)	
Divorced/separated/widowed	1,219 (3.7)	19,663 (15.6)	
Education			< 0.001
Middle school or lower	5,289 (11.9)	42,440 (28.3)	
High school	10,909 (53.1)	42,823 (49.8)	
College or higher	6,620 (35.0)	15,584 (22.0)	
Occupation			< 0.001
Experts	3,687 (19.7)	9,561 (12.5)	
Clerical/services roles	4,799 (23.9)	22,262 (24.2)	
Simple skilled roles	8,052 (24.7)	21,008 (13.5)	
Unemployed or housekeeper	6,236 (31.6)	47,924 (49.8)	
Monthly income (104 Korean won)			< 0.001
<1.0	5,939 (19.8)	30,838 (23.0)	
1.0 -<2.5	6,354 (25.3)	27,417 (25.7)	
2.5 -<4.0	5,329 (27.1)	22,446 (26.2)	
≥ 4.0	5,196 (27.9)	20,146 (25.1)	
Regular exercise	5,779 (22.1)	21,139 (17.8)	< 0.001
Regular alcohol drinker	2,759 (11.9)	2,905 (3.3)	< 0.001
Hypertension	3,436 (10.2)	19,476 (14.1)	< 0.001
Diabetes	1,326 (3.7)	6,084 (4.5)	< 0.001
Dyslipidemia	579 (2.0)	4,853 (4.4)	< 0.001
Secondhand smoke (hr/d)			< 0.001
None	12,484 (48.1)	42,417 (38.7)	
<1	4,537 (23.3)	36,999 (39.2)	
1-<3	400 (2.0)	1,667 (1.6)	
≥ 3	5,397 (26.6)	19,764 (20.4)	
Depressive symptoms			< 0.001
No	22,319 (97.6)	95,775 (94.5)	
Yes	493 (2.4)	5,030 (5.5)	
Diagnosed depression			< 0.001
No	22,603 (99.2)	97,964 (97.4)	
Yes	210 (0.8)	2,857 (2.6)	
Perceived stress			< 0.001
Minimal	5,119 (18.6)	19,372 (16.7)	
Mild	13,042 (58.8)	56,464 (57.6)	
Moderate	4,212 (20.5)	22,346 (22.9)	
Severe	431 (2.2)	2,583 (2.8)	

Values are presented as mean±standard deviation or number (%).

**Table 2.** Prevalence of mental health problems according to participant characteristics

Characteristics	Depressive symptoms			Diagnosed depression			High stress			
	No. of participants	No. with depressive symptoms	Prevalence (95% Cl)	No. of participants	No. with diagnosed depression	Prevalence (95% Cl)	No. of participants	No. with high stress	Prevalence (95% Cl)	
Gender										
Men	22,812	493	2.4 (2.2, 2.7)	22,813	210	0.8 (0.7,1.0)	22,804	4,643	22.7 (22.0, 23.4)	
Women	100,805	5,030	5.5 (5.3, 5.7)	100,821	2,857	2.6 (2.5, 2.7)	100,765	24,929	25.7(25.3, 26.1)	
Age (yr)										
19-29	17,657	715	4.2 (3.9, 4.6)	17,654	183	1.1 (0.9, 1.3)	17,656	4,483	26.0 (25.2, 26.7)	
30-39	21,373	874	4.2 (3.9, 4.5)	21,380	303	1.4(1.2, 1.6)	21,377	5,721	27.0 (26.3, 27.7)	
40-49	24,705	1,048	4.6 (4.3, 5.0)	24,709	476	1.7(16, 1.9)	24,705	6,136	25.9 (25.2, 26.5)	
50-59	24,873	1,194	5.3 (5.0, 5.7)	24,870	838	3.2 (2.9, 3.5)	24,869	5,794	23.3 (22.6, 24.0)	
60-69	18,583	959	6.5 (6.0, 7.0)	18,586	767	4.4 (4.1, 4.8)	18,582	4,214	23.5 (22.7, 24.3)	
≥70	16,426	733	5.3 (4.9, 5.8)	16,435	500	3.7 (3.2, 4.1)	16,380	3,224	20.5(19.8, 21.3)	
Marriage										
Never married	19,408	773	4.1 (3.8, 4.4)	19,404	231	1.2(10, 1.3)	19,404	4,971	26.3(25.6, 27.1)	
Married	83,257	3,441	4.6 (4.4, 4.8)	83,273	2,091	2.3 (2.1, 2.4)	83,261	19,900	24.6 (24.2, 25.0)	
Divorced/separated/widowed	20,866	1,302	7.2 (6.7, 7.6)	20,871	740	3.8 (3.5, 4.2)	20,819	4,676	24.5 (23.7, 25.3)	
Education										
Middle school or lower	47,703	2,446	6.3 (6.0, 6.7)	47,710	1,836	4.2 (4.0, 4.5)	47,652	11,134	24.9 (24.4, 25.5)	
High school	53,716	2,284	4.6 (4.4, 4.8)	53,721	980	1.7(16, 1.9)	53,718	12,935	24.7 (24.3, 25.2)	
College or higher	22,198	793	3.8 (3.5, 4.1)	22,203	251	1.2(10, 1.3)	22,199	5,503	25.7 (25.0, 26.4)	
Occupation										
Experts	13,245	445	3.5 (3.1, 3.9)	13,248	137	1.1 (0.9, 1.3)	13,248	3,679	29.2(28.3, 30.1)	
Clerical/services roles	27,054	1,099	4.5 (4.2, 4.8)	27,058	443	1.4(12, 1.6)	27,056	7,754	29.3 (28.6, 29.9)	
Simple skilled roles	29,052	1,084	4.2 (3.9, 4.5)	29,053	683	2.1 (1.9, 2.3)	29,049	6,506	23.8(23.1, 24.4)	
Unemployed or housekeeper	54,134	2,888	5.6 (5.4, 5.9)	54,140	1,803	3.0 (2.8, 3.2)	54,084	11,608	21.9(21.4, 22.4)	
Monthly income (104 Korean won)										
<1.0	36,759	2,003	6.2 (5.9, 6.6)	36,757	1,322	3.4 (3.2, 3.7)	36,720	8,984	26.4 (25.7, 27.0)	
1.0-<2.5	33,759	1,612	5.5 (5.2, 5.8)	33,765	863	2.6 (2.3, 2.8)	33,749	8,181	26.1 (25.5, 26.7)	
2.5-<4.0	27,765	1,047	4.2 (3.9, 4.5)	27,771	471	1.5(13, 1.7)	27,769	6,486	23.7(23.1, 24.3)	
≥ 4.0	25,334	861	3.6 (3.3, 3.9)	25,341	411	1.5(13, 1.7)	25,331	5,921	24.0 (23.4, 24.7)	
Secondhand smoke exposure (hr/d)										
None	54,883	1,879	3.7 (3.5, 3.9)	54,886	1,049	1.5(14, 1.6)	54,868	12,699	24.9 (24.4, 25.4)	
<1	41,522	2,117	5.2 (4.9, 5.5)	41,526	1,360	3.0 (2.8, 3.2)	41,486	8,794	21.6(21.1, 22.1)	
1-<3	2,066	125	5.7 (5.0, 6.4)	2,066	60	1.9(15, 2.3)	2,067	701	33.9(32.1, 35.7)	
≥ 3	25,146	1,402	6.2 (5.8, 6.5)	25,156	598	2.2 (2.0, 2.5)	25,148	7,378	30.1 (29.4, 30.8)	

**Table 3.** Association between SHSE and mental health problems in all participants

SHSE (hr/d)	Total	Depressive symptoms		Diagnosed depression		High stress	
		n (%)	OR (95% CI)	n (%)	OR (95% CI)	n (%)	OR (95% CI)
All participants							
None	54,883	1,879 (3.7)	1.00	1,049 (1.5)	1.00	12,699 (24.9)	1.00
<1	41,522	2,117 (5.2)	0.99 (0.88, 1.11)	1,360 (3.0)	1.15 (0.99, 1.34)	8,794 (21.6)	1.04 (0.98, 1.11)
1-<3	2,066	125 (5.7)	1.44 (1.14, 1.82)	60 (1.9)	1.11 (0.80, 1.54)	701 (33.9)	1.56 (1.38, 1.76)
≥ 3	25,146	1,402 (6.2)	1.59 (1.46, 1.74)	598 (2.2)	1.37 (1.20, 1.58)	7,378 (30.1)	1.33 (1.28, 1.40)
Men							
None	12,482	197 (1.7)	1.00	97 (0.7)	1.00	2,407 (21.9)	1.00
<1	4,537	126 (2.8)	0.99 (0.66, 1.48)	71 (1.4)	1.00 (0.52, 1.92)	748 (18.2)	1.06 (0.91, 1.25)
1-<3	399	14 (3.3)	2.01 (1.04, 3.86)	2 (0.6)	0.83 (0.19, 3.56)	118 (30.9)	1.59 (1.23, 2.04)
≥ 3	5,394	156 (3.2)	1.87 (1.43, 2.44)	40 (0.7)	1.06 (0.67, 1.69)	1,370 (27.4)	1.37 (1.25, 1.51)
Women							
None	42,401	1,682 (4.4)	1.00	952 (1.8)	1.00	10,292 (26.0)	1.00
<1	36,985	1,991 (5.6)	0.98 (0.87, 1.11)	1,289 (3.2)	1.16 (1.00, 1.36)	8,046 (22.2)	1.05 (0.98, 1.12)
1-<3	1,667	111 (6.5)	1.37 (1.07, 1.75)	58 (2.4)	1.14 (0.81, 1.59)	583 (35.0)	1.55 (1.35, 1.78)
≥ 3	19,752	1,246 (7.3)	1.56 (1.42, 1.72)	558 (2.8)	1.41 (1.22, 1.63)	6,008 (31.2)	1.32 (1.26, 1.39)

Adjusted for age, gender, body mass index, marital status, education level, occupation, monthly income, regular exercise, regular alcohol consumption, hypertension, diabetes, dyslipidemia, sleep time. SHSE, secondhand smoke exposure; OR, odds ratio; CI, confidence interval.

## Appendix 1.

Factors associated with secondhand smoke exposure (SHSE)

## Appendix 2.

Factors associated with diagnosed depression

## Appendix 3.

Factors associated with high stress

## Appendix 4.

Association between smoking status and mental health problems in total participants
