# Supplementary material for: Secondhand smoke exposure and mental health problems in Korean adults
Source: Epidemiol Health. 2016 Mar 14;38:e2016009. doi: 10.4178/epih.e2016009 (PMC4846743; doi:10.4178/epih.e2016009)
Supplement: Supplementary file 1 [file epih-38-e2016009-app1.pdf]

Appendix 1. Factors associated with secondhand smoke exposure (SHSE)

Variables (n = 123,665)	SHSE			Odds ratio (95% confidence interval)	
	No	Yes	p-value	Unadjusted	Gender, age -adjusted
Age (yr)			< 0.001		
19-39	16,702 (30.4)	22,343 (32.5)		1.00	1.00
40-59	24,001 (43.7)	25,586 (37.2)		0.85 (0.83, 0.89)	0.82 (0.79, 0.84)
≥ 60	14,198 (25.9)	20,865 (30.3)		1.67 (1.60, 1.73)	1.59 (1.53, 1.65)
Gender			< 0.001		
Men	12,484 (22.7)	10,334 (15.0)		1.00	1.00
Women	42,417 (77.3)	58,430 (85.0)		1.46 (1.41, 1.52)	1.43 (1.37, 1.48)
Marriage			< 0.001		
Never married	8,565 (15.6)	10,846 (15.8)		1.00	1.00
Married	37,593 (68.5)	45,692 (66.5)		1.00 (0.96, 1.03)	0.75 (0.71, 0.79)
Divorced/separated/widowed	8,701 (15.9)	12,181 (17.7)		1.20 (1.14, 1.26)	0.76 (0.71, 0.81)
Income per month (10^4 Korean won)			< 0.001		
≥ 4.0	12,413 (22.6)	12,929 (18.8)		1.00	1.00
< 1.0	16,088 (29.3)	20,689 (30.1)		1.53 (1.47, 1.60)	1.46 (1.40, 1.53)
1.0- < 2.5	14,487 (26.4)	19,284 (28.0)		1.42 (1.36, 1.49)	1.40 (1.34, 1.46)
2.5- < 4.0	11,913 (21.7)	15,862 (23.1)		1.28 (1.23, 1.34)	1.28 (1.22, 1.34)
Education			< 0.001		
Middle school or lower	20,501 (37.3)	27,228 (39.6)		1.00	1.00
High school	21,968 (40.0)	31,764 (46.2)		0.83 (0.80, 0.86)	0.81 (0.78, 0.85)
College or higher	12,432 (22.6)	9,772 (14.2)		0.41 (0.40, 0.43)	0.41 (0.39, 0.43)
Occupation			< 0.001		
Unemployed or housekeeper	8,023 (14.6)	46,137 (67.2)		1.00	1.00
Experts	10,129 (18.5)	3,119 (4.5)		0.05 (0.04, 0.05)	0.04 (0.04, 0.05)
Clerical/services	16,823 (30.7)	10,238 (14.9)		0.08 (0.08, 0.09)	0.08 (0.08, 0.08)
Simple skill	19,855 (36.2)	9,205 (13.4)		0.09 (0.09, 0.10)	0.09 (0.09, 0.10)
Drinking			< 0.001		
Regular alcohol drinkers	2,710 (4.9)	2,954 (4.3)		0.83 (0.78, 0.89)	0.95 (0.88, 1.01)

Values are presented as number (%).
