# Supplementary material for: Secondhand smoke exposure and mental health problems in Korean adults
Source: Epidemiol Health. 2016 Mar 14;38:e2016009. doi: 10.4178/epih.e2016009 (PMC4846743; doi:10.4178/epih.e2016009)
Supplement: Supplementary file 2 [file epih-38-e2016009-app2.pdf]

Appendix 2. Factors associated with diagnosed depression

Variables (n = 123,665)	Diagnosed depression			Odds ratio (95% confidence interval)	
	No	Yes	p-value	Unadjusted	Gender, age -adjusted
Age (yr)			< 0.001		
19-39	38,548 (32.0)	486 (15.9)		1.00	1.00
40-59	48,265 (40.0)	1,314 (42.8)		1.99 (1.76, 2.26)	1.82 (1.61, 2.07)
≥ 60	33,754 (28.0)	1,267 (41.3)		3.46 (3.03, 3.94)	3.13 (2.75, 3.57)
Gender			< 0.001		
Men	22,603 (18.8)	210 (6.9)		1.00	1.00
Women	97,964 (81.3)	2,857 (93.2)		3.17 (2.63, 3.81)	2.74 (2.28, 3.29)
Marriage			< 0.001		
Never married	19,173 (15.9)	231 (7.5)		1.00	1.00
Married	81,182 (67.4)	2,091 (68.3)		1.96 (1.66, 2.31)	0.93 (0.77, 1.12)
Divorced/separated/widowed	20,131 (16.7)	740 (24.2)		3.37 (2.80, 4.05)	1.02 (0.80, 1.30)
Income per month (10^4 Korean won)			< 0.001		
≥ 4.0	24,930 (20.7)	411 (13.4)		1.00	1.00
< 1.0	35,435 (29.4)	1,322 (43.1)		2.38 (2.07, 2.73)	1.71 (1.47, 1.99)
1.0- < 2.5	32,902 (27.3)	863 (28.1)		1.76 (1.53, 2.03)	1.59 (1.37, 1.83)
2.5- < 4.0	27,300 (22.6)	471 (15.4)		1.02 (0.87, 1.20)	1.01 (0.85, 1.19)
Education			< 0.001		
Middle school or lower	45,874 (38.1)	1,836 (59.9)		1.00	1.00
High school	52,741 (43.7)	980 (32.0)		0.40 (0.36, 0.44)	0.58 (0.50, 0.67)
College or higher	21,952 (18.2)	251 (8.2)		0.26 (0.22, 0.31)	0.42 (0.34, 0.51)
Occupation			< 0.001		
Unemployed or housekeeper	52,337 (43.5)	1,803 (58.8)		1.00	1.00
Experts	13,111 (10.9)	137 (4.5)		0.36 (0.29, 0.43)	0.52 (0.42, 0.64)
Clerical/services	26,615 (22.1)	443 (14.5)		0.46 (0.40, 0.53)	0.59 (0.51, 0.67)
Simple skill	28,370 (23.6)	683 (22.3)		0.70 (0.62, 0.79)	0.78 (0.69, 0.89)
Drinking			0.004		
Regular alcohol drinkers	5,557 (4.61)	107 (3.49)		0.69 (0.55, 0.87)	1.10 (0.87, 1.39)

Values are presented as number (%).
