# Supplementary material for: Secondhand smoke exposure and mental health problems in Korean adults
Source: Epidemiol Health. 2016 Mar 14;38:e2016009. doi: 10.4178/epih.e2016009 (PMC4846743; doi:10.4178/epih.e2016009)
Supplement: Supplementary file 3 [file epih-38-e2016009-app3.pdf]

Appendix 3. Factors associated with high stress

Variables (n = 123,665)	High stress			Odds ratio (95% confidence interval)	
	No	Yes	p-value	Unadjusted	Gender, age -adjusted
Age (yr)			< 0.001		
19-39	28,829 (30.7)	10,204 (34.5)		1.00	1.00
40-59	37,644 (40.1)	11,930 (40.3)		0.91 (0.88, 0.94)	0.89 (0.86, 0.92)
≥60	27,524 (29.3)	7,438 (25.2)		0.79 (0.76, 0.83)	0.76 (0.74, 0.81)
Gender			< 0.001		
Men	18,161 (19.3)	4,643 (15.7)		1.00	1.00
Women	75,836 (80.7)	24,929 (84.3)		1.18 (1.13, 1.23)	1.23 (1.17, 1.28)
Marriage			< 0.001		
Never married	14,433 (15.4)	4,971 (16.8)		1.00	1.00
Married	63,361 (67.5)	19,900 (67.4)		0.91 (0.88, 0.95)	1.03 (0.98, 1.08)
Divorced/separated/widowed	16,143 (17.2)	4,676 (15.8)		0.91 (0.86, 0.96)	1.10 (1.02, 1.19)
Income per month (10^4 Korean won)			< 0.001		
≥4.0	19,410 (20.7)	5,921 (20.0)		1.00	1.00
< 1.0	27,736 (29.5)	8,984 (30.4)		1.13 (1.08, 1.19)	1.24 (1.17, 1.30)
1.0- < 2.5	25,568 (27.2)	8,181 (27.7)		1.12 (1.07, 1.17)	1.14 (1.09, 1.20)
2.5- < 4.0	21,283 (22.6)	6,486 (21.9)		0.98 (0.94, 1.03)	0.98 (0.93, 1.03)
Education			0.050		
Middle school or lower	36,518 (38.9)	11,134 (37.7)		1.00	1.00
High school	40,783 (43.4)	12,935 (43.7)		0.99 (0.95, 1.03)	0.80 (0.76, 0.84)
College or higher	16,696 (17.8)	5,503 (18.6)		1.04 (0.99, 1.09)	0.86 (0.81, 0.91)
Occupation			< 0.001		
Unemployed or housekeeper	42,476 (45.2)	11,608 (39.3)		1.00	1.00
Experts	9,569 (10.2)	3,679 (12.5)		1.47 (1.40, 1.55)	1.47 (1.40, 1.55)
Clerical/services	19,302 (20.6)	7,754 (26.2)		1.47 (1.42, 1.54)	1.46 (1.40, 1.52)
Simple skill	22,543 (24.0)	6,506 (22.0)		1.11 (1.06, 1.16)	1.18 (1.13, 1.24)
Drinking			< 0.001		
Regular alcohol drinkers	3,978 (4.23)	1,683 (5.69)		1.40 (1.31, 1.50)	1.46 (1.36, 1.57)

Values are presented as number (%).

Appendix 4. Association between smoking status and mental health problems in total participants

Smoking status	Total	Depressive symptoms		Diagnosed depression		High stress	
Total participants							
Non-smokers	123,617	5,523 (4.8)	1.00	3067 (2.2)	1.00	29,572 (25.0)	1.00
Former smokers	31,988	961 (3.4)	1.58 (1.41, 1.78)	466 (1.4)	1.81 (1.52, 2.15)	6,982 (24.7)	1.21 (1.15, 1.27)
Current smokers	44,491	1,807 (4.3)	1.92 (1.73, 2.14)	582 (1.3)	1.89 (1.59, 2.26)	14,099 (34.5)	1.75 (1.67, 1.83)
Men							
Non-smokers	22,812	493 (2.4)	1.00	210 (0.8)	1.00	4,643 (22.7)	1.00
Former smokers	29,962	758 (2.9)	1.31 (1.12, 1.53)	341 (1.1)	1.36 (1.06, 1.74)	6,301 (24.0)	1.13 (1.07, 1.19)
Current smokers	41,447	1,373 (3.6)	1.58 (1.38, 1.81)	347 (0.8)	1.16 (0.92, 1.47)	12,775 (33.8)	1.63 (1.55, 1.72)
Women							
Non-smokers	100,805	5,030 (5.5)	1.00	2,857 (2.6)	1.00	24,929 (25.7)	1.00
Former smokers	2,026	203 (10.4)	1.70 (1.42, 2.04)	125 (5.3)	1.88 (1.48, 2.39)	681 (33.8)	1.42 (1.26, 1.60)
Current smokers	3,044	434 (15.7)	2.55 (2.22, 2.93)	235 (8.2)	2.95 (2.47, 3.54)	1,324 (45.5)	2.13 (1.93, 2.34)

Values are presented as number (%) or odds ratio (95% confidence interval).
Adjusted for age, gender, body mass index, marital status, education level, occupation, income, regular exercise, alcohol drinking, hypertension, diabetes, dyslipidemia, sleep time.
